# The Effect of Post-Weld Hot-Rolling on the Properties of Explosively Welded Mg/Al/Ti Multilayer Composite

**DOI:** 10.3390/ma13081930

**Published:** 2020-04-19

**Authors:** Marcin Wachowski, Robert Kosturek, Lucjan Śnieżek, Sebastian Mróz, Andrzej Stefanik, Piotr Szota

**Affiliations:** 1 Faculty of Mechanical Engineering, Military University of Technology, 00-908 Warsaw, Poland; robert.kosturek@wat.edu.pl (R.K.); lucjan.sniezek@wat.edu.pl (L.Ś.); 2 Faculty of Production Engineering and Materials Technology, Czestochowa University of Technology, 42-201 Częstochowa, Poland; mroz.sebastian@wip.pcz.pl (S.M.); stefanik.andrzej@wip.pcz.pl (A.S.); szota.piotr@wip.pcz.pl (P.S.)

**Keywords:** explosive welding, light alloys, hot rolling, composite, microstructure, intermetallic

## Abstract

The paper describes an investigation of an explosively welded Mg/Al/Ti multilayer composite. Following the welding, the composite was subjected to hot-rolling in three different temperatures: 300 °C, 350 °C and 400 °C, with a total relative strain of 30%. The rolling speed was 0.2 m/s. The investigation of the composite properties involves microhardness analysis and mini-specimen tensile tests of the joints. The composite Mg/Al and Al/Ti bonds in the as-welded state and after rolling in 400 °C were subjected to microstructure analysis using scanning electron (SEM) and transmission electron microscopy (TEM). In the Al/Ti interface, the presence of melted zones with localized intermetallic precipitates has been reported in the as-welded state, and it has been stated that hot-rolling results in precipitation of intermetallic particles from the melted zone. The application of the hot-rolling process causes the formation of a continuous layer in the Mg/Al joint, consisting of two intermetallic phases, Mg_2_Al_3_ (β) and Mg_17_Al_12_ (γ).

## 1. Introduction

Light alloy multilayer composites are very promising materials, especially in military applications, due to their high specific strength and ballistic resistance. One of the most interesting is Mg/Al/Ti laminate, characterized by an increasing gradient of hardness. Manufacturing of this material is very problematic, considering the significant differences in properties of components to be joined, such as melting point and ductility. An additional factor is the tendency of the formation of intermetallic compounds between Al/Ti and Mg/Al interfaces [1,2,3,4,5,6,7,8]. Despite the good mechanical properties of intermetallic phases, their brittleness causes further problems during the plastic forming of multilayer composite, including a risk of delamination. An effective method to obtain the Mg/Al/Ti multilayer composite is explosive welding, which is a solid state welding process. Solid state welding of multilayer materials has advantages in accordance with fusion-based welding, i.e., shorter time of process and limited intermetallic formation at the bond zone [9,10]. In the explosive welding process, the energy released during detonation of the high explosive is used to accelerate one metal plate into another, and as a result, the high velocity collision of metal plates occurs, which brings the surfaces of the colliding metals close enough to each other to obtain interaction between their atoms and make the formation of a metallic bond between them possible. The main advantages of this process are the possibility of joining elements of large sizes and of a wide range of base and cladding material thickness without formation of a diffusion zone, which is a potential area of intermetallic compounds presence. Compared to other welding methods, impact welding allows one to obtain high-efficiency joints between a wide variety of similar and/or dissimilar metals without forming intermetallic phases in the bond zones. In the literature, the microstructure and mechanical properties of tri-metal, Ti/Al and Mg/Al layered composites obtained by various methods are widely described [11,12,13,14,15,16]. Zhang et al. revealed the interface microstructure of explosively welded Ti Gr.2/AA6061/AZ31B composite [17]. A periodic wavy bond without melted zones was observed between Ti and Al plates. A larger wavy interface was revealed in the Mg/Al joint. Fouad presented research results of Ti/AA6081/AZ31 obtained by hot isostatic pressing [18]. An intermetallic layer with a maximum thickness of 10 µm was formed at the AA6081/AZ31 interface and the presence of an Al_2_O_3_ interface was confirmed. Motevalli and Eghbali investigated the AA1050/cp-Ti/AZ31 laminated composite obtained by accumulative roll bonding [19]. Optimum mechanical properties and microstructural characteristics were achieved after four deformation passes with the strength of the composite at 335.9 MPa. Research results of Wu et al. on Mg/AA5052 laminate fabricated by accumulative roll bonding (ARB) revealed that after the final three cycles of rolling, cracking of the coarse Mg/Al intermetallic compound and rupture of the Al layers occurred [20]. Research on diffusion-welded Mg/Al joints revealed the presence of Al_12_Mg_17_/Al_3_Mg_2_ intermetallic compounds [21]. Fronczek et al. revealed that annealing of explosively welded A1050/AZ31/A1050 composite plate results in the formation of the Mg_2_Al_3_ intermetallic phase localized near to the A1050 plate, Mg_17_Al_12_, closer to the AZ31 clad and the minor amount of Mg_23_Al_30_ [22]. Lazurenko et al. investigated the multilayer cp-Ti/Al-1Mn aluminum alloy composite produced by explosive welding and revealed the formation of Al_3_Ti and AlTi phases, metastable phase Al_5_Ti_3_, disordered AlTi_3_, amorphous structures and ordered solid solutions [23]. Although explosive welding allows to significantly limit the formation of intermetallic compounds on a Mg/Al/Ti multilayer material interface, the composite itself is subjected to the further technological operations after welding; usually plastic forming (including hot forming) in order to obtain a specific shape [24]. The application of hot forming after the explosive welding process can result in the formation of intermetallic phases in the considered bond zones that influence the mechanical properties of the whole composite. While the microstructure and mechanical properties of laminated composites obtained by explosive welding are widely described in the literature, information about research on the Mg/Al/Ti laminate, obtained by the method of explosive welding with subsequent hot-forming, is not presented. Therefore, it is important to understand the effect of the post-weld hot-forming process on the microstructure, especially the formation of diffusion zones. The aim of this paper is to investigate the influence of post-weld hot-rolling on the properties of the composite, especially the Mg/Al and Al/Ti interfaces, which are the potential areas of intermetallic compounds formation.

## 2. Materials and Methods

**Figure 1:**
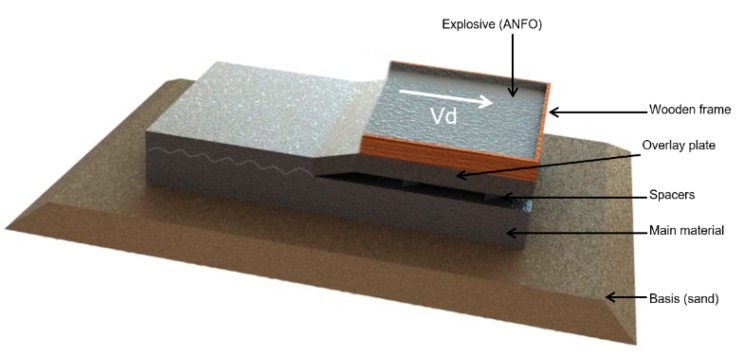
Scheme of explosive welding process, Vd—detonation velocity.

For the research, a Mg/Al/Ti multilayer composite was produced by explosive welding. The process of explosive welding was carried out in the company ZTW EXPLOMET sp.j. Gałka, Szulc (Opole, Poland). The plates used to obtain the investigated composite had dimensions equal to 1200 × 800 mm. The type of explosive used during the process was ANFO (ammonium nitrate fuel oil). The thickness of the explosive layer was 50 mm, the stand-off distances for both Al/Ti and Mg/Al were 5 mm and the initial position angle between plates was 0°. Detonation velocity was equal to 3200 m/s. Before joining, surfaces of the joined materials were polished and cleaned by acetone. The charge initiation point was located in the center of the shorter side of the plate. The schematic of the explosive connection system is shown in Figure 1.

**Figure 2:**
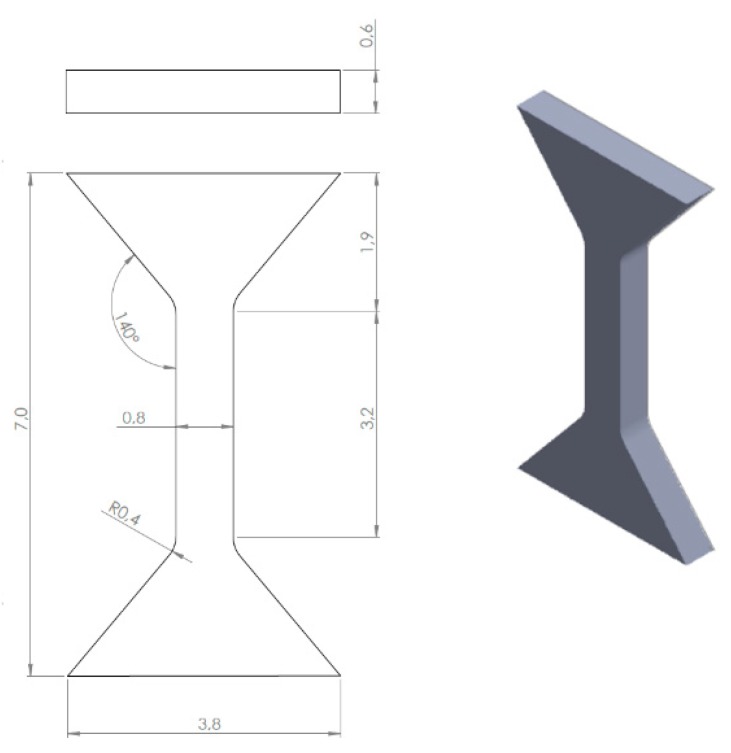
The scheme of mini-specimen for tensile test.

Obtaining an AZ31/AA2519/Ti6Al4V laminate requires the use of an additional AA1050 aluminum layer between AZ31/AA2519 and AA2519/Ti6Al4V plates. The following plates were used to make the laminate: magnesium alloy AZ31 (5 mm thick), aluminum alloy AA2519 cladded on both sides with AA1050 (4 mm thick in total) and titanium alloy Ti6Al4V (5 mm thick). The chemical composition of the components is shown in Table 1. The chemical composition of the materials was established by the materials supplier. The multilayer material was investigated in the state after the explosive welding and after applying the forming process—hot rolling, performed in three different temperatures: 300 °C, 350 °C and 400 °C, with a total relative strain of 30%. For laboratory testing, a laboratory rolling mill with a working roll diameter of 300 mm was used. The rolling speed was 0.2 m/s. The investigation of the composite properties involves a microhardness distribution analysis, performed with load of 0.98 N was applied for 10 s, and mini-specimen tensile testing conducted on Instron 8802 MTL universal testing machine (Instron, Warsaw, Poland). Space between microhardness test indentations was equal to 150 µm. The scheme of the used mini-specimen is presented in Figure 2. The tensile strength was measured separately for Ti6Al4V/AA1050/AA2519 and AZ31/AA1050/AA2519 joints. For obtaining the tensile strength values, 3 mini-samples of Ti6Al4V/AA1050/AA2519 material for each state (as-welded, hot-rolled 300 °C, 350 °C, 400 °C) were tested (equal to 12 samples). The same case was for AZ31/AA1050/AA2519 mini-samples (equal to 12 samples as well). The samples were cut from the central part of the plate in order to avoid the delaminated or weakened areas of the joint that are characteristic of the edges and corners of the plates being joined. In addition, the test plates were cut at a certain angle to each other, as the impact wave generated by the explosion propagated radially through the material. Therefore, for a representative microstructure, the test plates should be cut in a direction parallel to the direction of the impact wave propagation.

The composite Mg/Al and Al/Ti bonds in the as-welded state and after rolling at 400 °C were subjected to microstructure analysis using scanning electron (SEM) (JEOL, Warsaw, Poland) and transmission electron microscopy (TEM) (JEOL, Warsaw, Poland). Microscopic examinations were carried out on samples whose surfaces were oriented parallel to the direction of propagation of the shock wave during the joining process and parallel to the rolling direction. A wire EDM was used to cut the samples. High cutting accuracy was achieved, and the heat of cut was avoided on the surface layer of the materials. Because the hot rolling process can contribute to significant changes in the microstructure, not only of the magnesium alloy but also of aluminum and titanium, and can also cause significant structural changes within the bond zone, to understand the phenomena occurring during explosive bonding and hot rolling, advanced microscopic methods were used. In order to visualize the microstructure of the composite components and the morphology of the transition layers, the obtained material was investigated using scanning electron microscope JEOL JSM-6610 equipped with an X-ray energy dispersion (EDX) spectrometer and backscattered electron detector (BSE) (JEOL, Warsaw, Poland). As a part of metallographic sample preparation, samples were cut along the axial direction using a precision diamond saw and then mounted in resin, ground with the abrasive paper of 80, 320, 600, 1200, 2400 and 4000 gradations and polished using diamond pastes (3 µm and 1 µm gradation). In selected joints, a precise microstructural analysis was carried out. For this purpose, samples were cut for observation on the transmission electron microscope JEOL JEM-1200. The identification of phases present in individual layers of multilayer material was carried out using the electron diffraction technique (SAED) (JEOL, Warsaw, Poland). A light microscopy investigation was performed using Olympus LEXT OLS4100 microscope (OLYMPUS, Warsaw, Poland).

## 3. Results and Discussion

**Figure 3:**
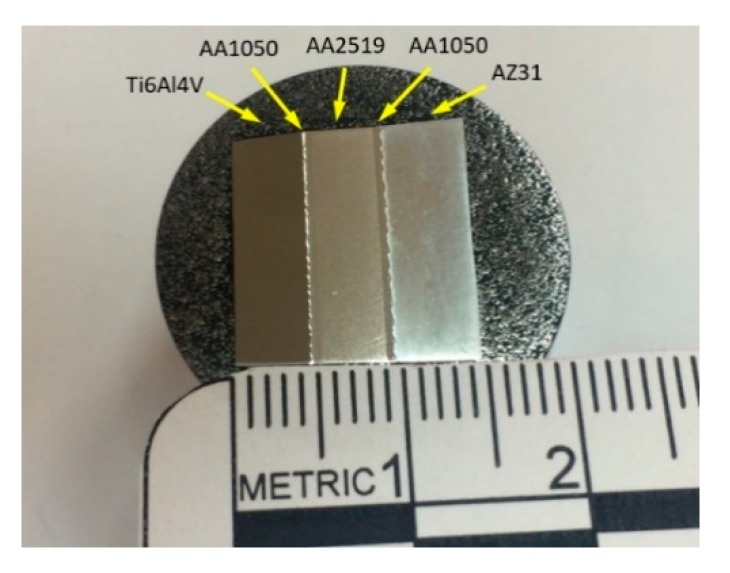
Cross-section of the obtained multilayer composite.

After the joining process, the AZ31/AA1050/AA2519/AA1050/Ti6Al4V multilayer composite was obtained (Figure 3).

In this investigation, the four different samples have been taken under investigation: one in the as-welded state and three after hot-rolling at different temperatures. The designation of samples, together with their descriptions, are presented in Table 2.

**Figure 4:**
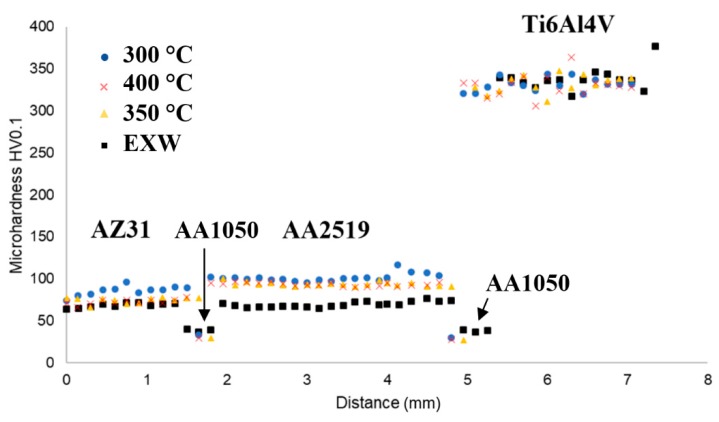
The distribution of microhardness in the investigated composite in the as-welded state (EXW) and after hot-rolling (300C, 350C, 400C).

In the first part of the investigation, the microhardness measurements were performed in order to establish the microhardness distribution for each sample (Figure 4). It is note-worthy that in the bonded areas in some cases it is difficult to obtain an accordance with each layer in all samples; this is an effect of small differences in the width of materials after the explosive welding process. The material affected by this phenomenon in the most visible way is the AA1050 interlayer, due to its softness. As can observed in the microhardness distribution, the hot-rolling process increases the microhardness for AZ31 and AA2519 components. The total relative strain was constant (30%), and only the rolling temperature was a factor which influenced the final properties of the composite. In the case of the 300C sample, it can be stated that the highest value of strain hardening was obtained as the result of the post-weld hot-rolling process. Rolling at a higher temperature of 350C and 400C results in slightly lower values of microhardness for both AZ31 and AA2519. This is probably the result of involving recrystallization processes in the higher temperature range [25,26]. Ti6Al4V is characterized by the relatively higher spread of microhardness values and this tendency maintains after applying of hot-rolling, due to the high recrystallization temperature of this alloy [27]. The highest strain hardening of AZ31 and AA2519 has been achieved after rolling at 300 °C, with reported values of microhardness of about 80 HV0.1 and 105 HV0.1, respectively. For the titanium component, the spread of results covers values from 300 HV0.1 to 370 HV0.1.

**Figure 5:**
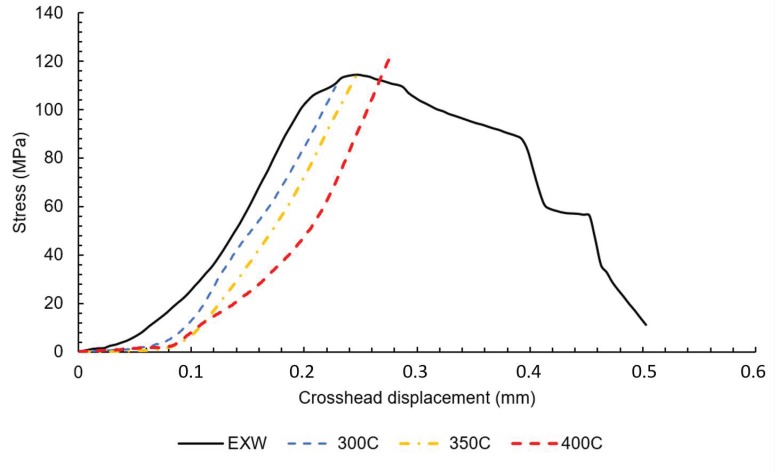
The tensile curves for Mg/Al interface in the as-welded state (EXW) and after hot-rolling (300C, 350C, 400C).

**Figure 6:**
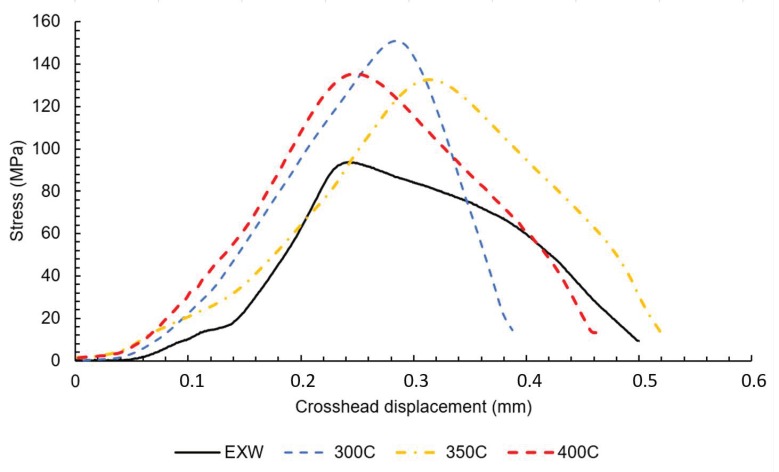
The tensile curves for Al/Ti interface in the as-welded state (EXW) and after hot-rolling (300C, 350C, 400C).

Now, we establish the basic mechanical properties that the tensile tests of mini-specimens were carried on. Mini-specimens were cut from Mg/Al and Al/Ti joints separately for all analyzed samples. The obtained results of the tensile test for Mg/Al and Al/Ti joints are presented in Figure 5 and Figure 6, respectively. A summary of the tensile tests and the standard deviation of mechanical properties is presented in Table 3.

**Figure 7:**
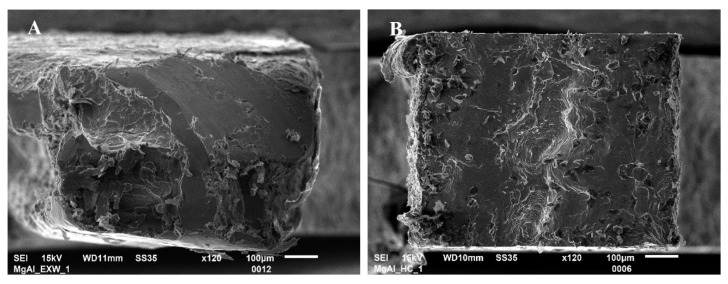
The fracture surface of Mg/Al mini-specimen in the as-welded state (**A**) and after hot-rolling at 400 °C (**B**).

The analysis of the obtained curves allows one to draw a conclusion that post-weld hot rolling affects both joints in a different way. The Mg/Al interface shows a strong correlation of tensile strength and rolling temperature. The sample in the as-welded state is characterized by tensile strength about 114 MPa. The curves obtained for samples after hot-rolling are characterized by the lack of plastic deformation. It has to be taken into consideration that the higher value of elongation for the as-welded sample could not be an effect of plastic deformation but also crack opening or delamination. Although hot-rolling caused a noticeable decrease in plasticity, the post-weld processing at 400 °C given a positive effect in the highest tensile strength of 125 MPa. At the same time, the Al/Ti interface is characterized by significant differences in tensile strength, with 101 MPa value reported for sample in the as-welded state, and 154 MPa for the sample after hot-rolling in 300 °C. The samples 350 °C and 400 °C have intermediate values of tensile strength—132 MPa and 135 MPa, respectively. In Ti6Al4V/AA1050/AA2519 mini-samples independently on the state of the material, the failure occurs on Ti6Al4V/AA1050 interface, which indicates the crucial role of this bond in the composite cohesion. The Mg/Al fracture surfaces of samples EXW (Figure 7A) and 400C (Figure 7B) are presented below.

**Figure 8:**
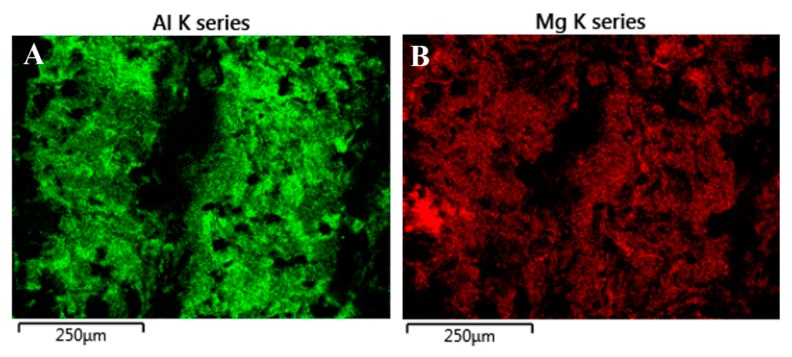
The results of the element distribution on the fracture surface of Mg/Al mini-specimen after hot-rolling at 400 °C: aluminum (**A**) and magnesium (**B**).

The fractography investigation revealed the brittle mechanism of failure for samples before and after hot-rolling. However, the failure of material after treatment was proceeded through the diffusion layer, which was confirmed by EDX investigation. The results of the element distribution on the fracture surface of Mg/Al mini-specimen after hot-rolling at 400 °C for aluminum (Figure 8A) and magnesium alloy (Figure 8B) are presented below. Obtained maps show that the surface of the fractured sample is characterized by the presence of both aluminum and magnesium. This result suggests that failure occurs in the diffusion layer. In all AZ31/AA1050/AA2519 mini-samples independently on the state of the material, the failure occurs on the AZ31/AA1050 interface, which indicates on lower strength properties of AZ31/AA1050 than AA1050/AA2519 independently on the presence of thick or thin intermetallic phase layer [28].

**Figure 9:**
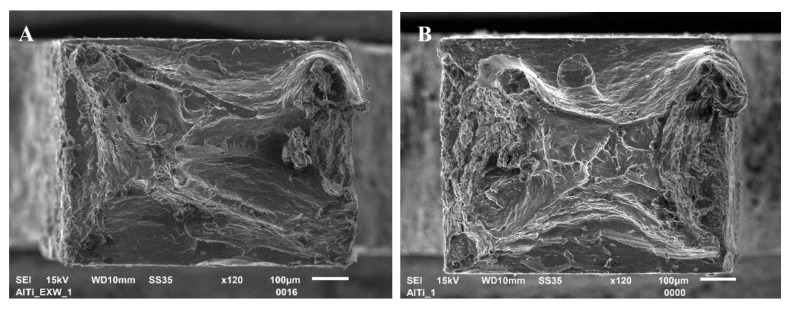
The fracture surface of Al/Ti mini-specimen in the as-welded state (**A**) and after hot-rolling at 400 °C (**B**).

The Al/Ti fracture surfaces of samples EXW (Figure 9A) 400C (Figure 9B) are presented below. The analysis of the obtained fracture surfaces shows that despite the similar character of fracture, the sample subjected to the hot-rolling exhibits a slightly higher participation of ductile fracture.

**Figure 10:**
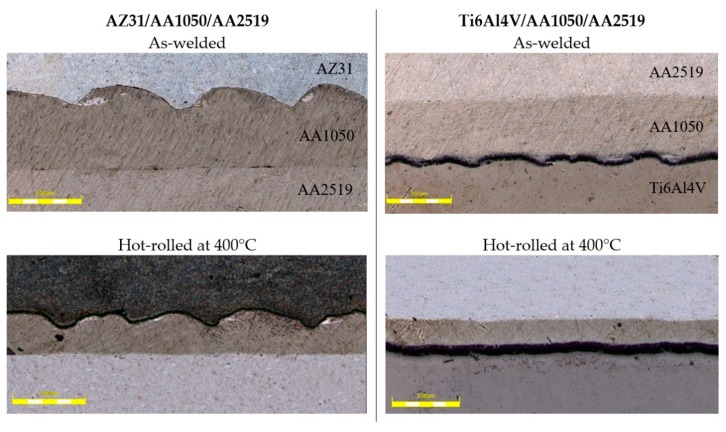
Light microscopy images of investigated interfaces (500 µm scale bar).

For microstructure analysis, two samples have been chosen: in the as-welded state and after hot-rolling at 400 °C. Light microscopy images of investigated interfaces are presented in Figure 10.

**Figure 11:**
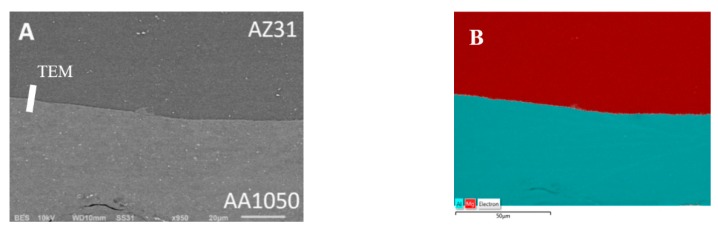
AZ31/AA1050 joint in as-welded state: (**A**) SEM results, (**B**) Mg and Al elements distribution on the sample surface.

The AZ31/AA1050 joint is a potential area of intermetallic phase growth from the Al-Mg system. Analyzing the phase equilibrium system of the main alloying elements of the considered joint, three intermetallic compounds can be distinguished: Mg_2_Al_3_ (β), Mg_17_Al_12_ (γ) and Mg_23_Al_30_ (R or ε). An explosive welding process can result in the formation of vortex in the joint line, characterized by the local mixing of joint materials [29]. Similarly to the bimetallic joint, the vortex is also the area with potential for intermetallic growth. In the AZ31/AA150 joint not subjected to thermo-plastic treatment, no formation of intermetallic phases was observed, which was confirmed by the results of SEM tests (Figure 11A) and analysis of the Mg and Al elements distribution on the sample surface—EDX (Figure 11B). An observed flat joint was observed free of any defects in the form of voids, delamination and melted areas, which proves that the joining process was carried out correctly.

**Figure 12:**
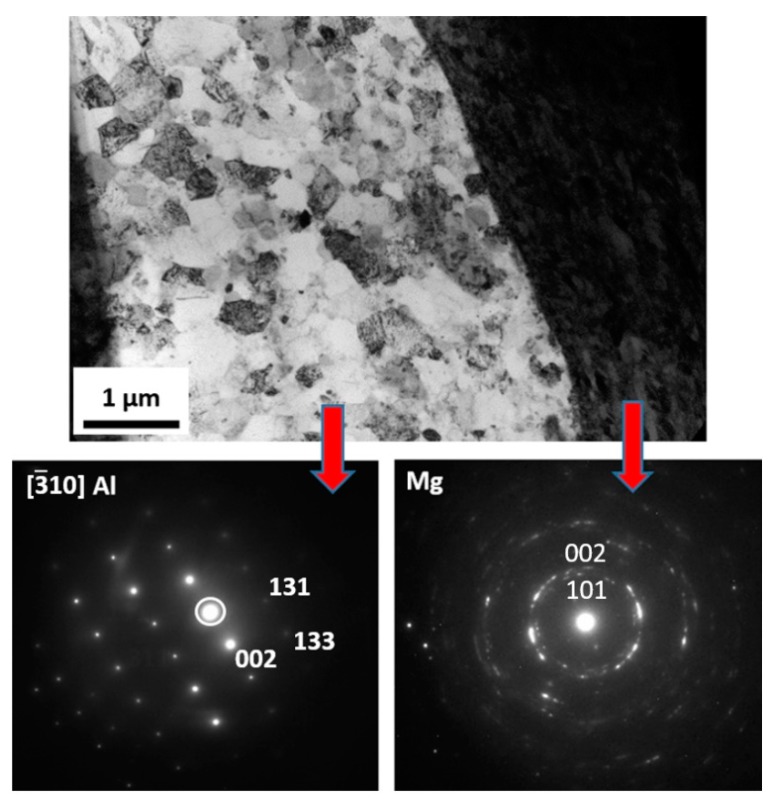
TEM image of AZ31/AA1050 joint microstructure in as-welded state with selected area diffraction patterns (SAED). TEM lamella position in Figure 11A.

In order to confirm the absence of intermetallic phases and melted zones at the Mg-Al boundary, a phase analysis was carried out using the SAED electron diffraction technique (Figure 12). The TEM results also revealed the occurrence of high plastic deformation in the AZ31 material near the joint and the presence of equiaxial ultrafine grains in the microstructure of AA1050 material with their size about 1 µm.

**Figure 13:**
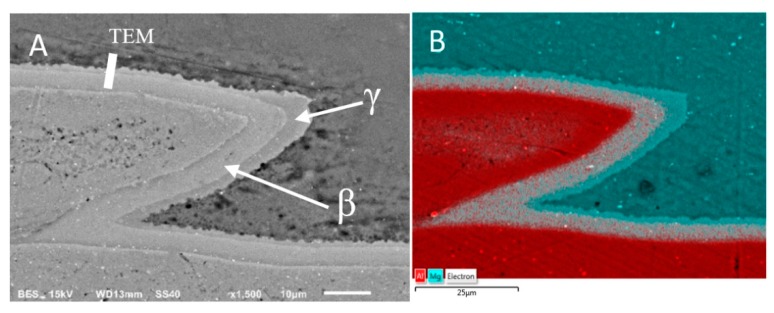
Microstructure of AZ31/AA1050 joint after hot-rolling in 400 °C: (**A**) SEM results, (**B**) Mg and Al elements distribution on the sample surface.

**Figure 14:**
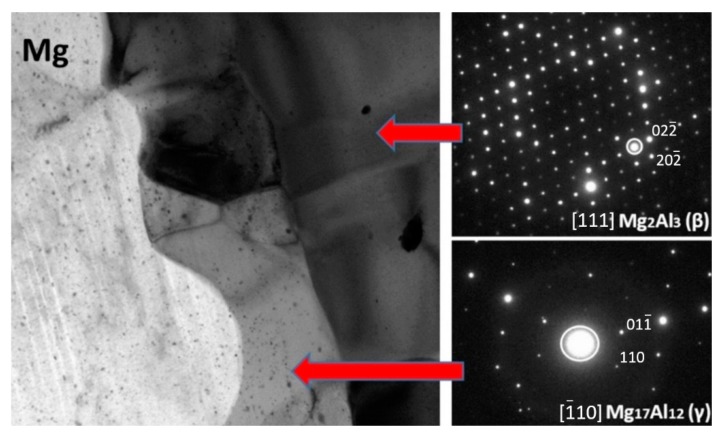
TEM image of AZ31/AA1050 joint microstructure after hot-rolling at 400 °C with selected area diffraction patterns (SAED). TEM lamella position at Figure 13A.

Scanning and transmission electron microscopy investigation of the AZ31/AA1050 joint after the rolling process allows one to conclude that, due to the diffusion of aluminum and magnesium in the joint area during hot rolling, a continuous layer consisting of two intermetallic phases is formed. The microstructure of the characteristic joint zone is presented in Figure 13. Due to the short time of the explosive welding process and small zone impact, the cooling rates of joined materials allowed one to limit the formation of the intermetallic compounds. At the same time, the hot rolling process promotes diffusion changes within the joints, which may result in the formation of intermetallic phases. The severe plastic deformations of the joint zone decreases the amount of energy necessary to initiate heat-activated phenomena, including the formation of new compounds. In the case of the Mg/Al joint, the formed diffusion zone had a layered character with two sublayers of different compounds. From the aluminum alloy side, the Mg_2_Al_3_ (β) phase is formed and the Mg_17_Al_12_ (γ) phase from the magnesium alloy was confirmed by TEM investigation results (Figure 14). The results indicated the significant influence of the hot rolling process on the intermetallic compounds formation on the joint interface.

It should also be noted that there are disproportions in the thickness of the individual intermetallic phases (maximum 4.5 µm for β and 9 µm for γ), resulting in differences in their growth rates (Figure 13). In order to confirm the presence of intermetallic phases β and γ at the Mg-Al limit, a phase analysis was performed using the SAED electron diffraction technique (Figure 14). The higher-resolution observation revealed a lack of cracks or delamination between intermetallic layers and base materials. The TEM investigation revealed coarsened equiaxed grains in the microstructure of AZ31.

**Figure 15:**
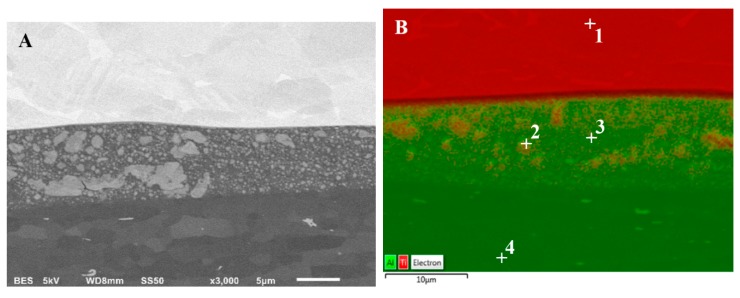
Microstructure of AA1050/Ti6Al4V joint in as-welded state: (**A**) SEM results, (**B**) Ti and Al elements distribution on the sample surface.

**Figure 16:**
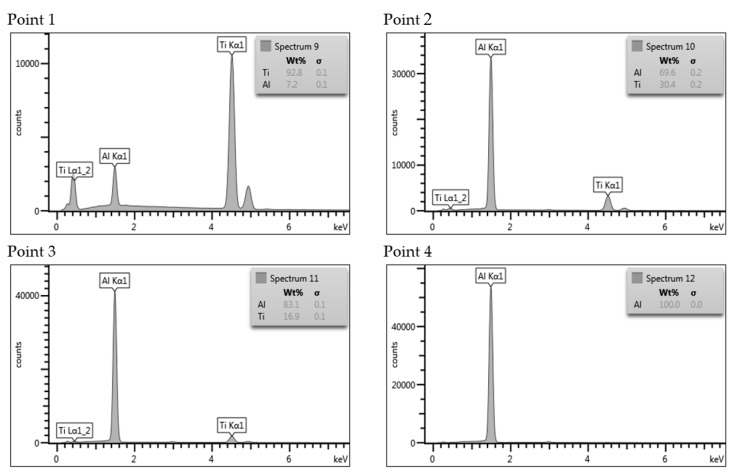
Element compositions corresponding to the spectrums in Figure 15B.

Similarly, as in the case of Al/Mg bonds, the joint AA1050/Ti6Al4V is also a potential area of intermetallic phase growth. Analyzing the titanium-aluminum phase balance system, four intermetallic compounds can be identified: Ti_3_Al (α2), TiAl (γ), TiAl_2_ and TiAl_3_. In contrast to the stoichiometric TiAl_3_ and TiAl_2_ phases, the Ti_3_Al and TiAl phases are present in a wide range of aluminum contents. The research results of the as-welded AA1050/Ti6Al4V joint revealed the presence of the melted zone with localized intermetallic particles (Figure 15A). The analysis of Ti and Al elements distribution on the sample surface—EDX allows to observe the mixture character of the melted zone (Figure 15B). Element compositions corresponding to the spectrums in Figure 15B are presented in Figure 16.

**Figure 17:**
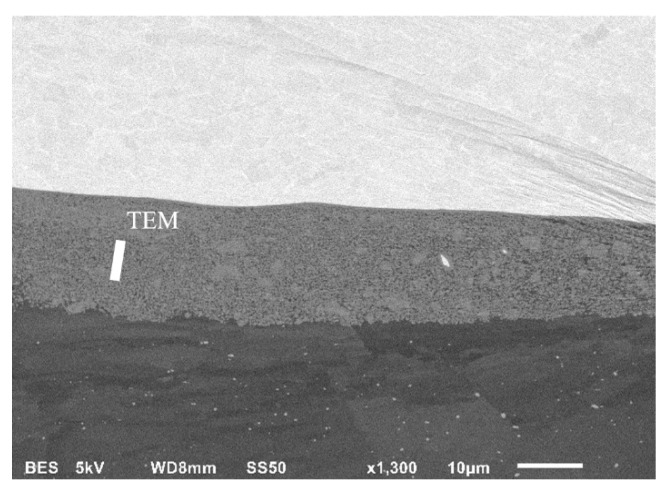
SEM image of the microstructure of AA1050/Ti6Al4V joint hot-rolled at 400 °C.

The results of the research on the material after hot rolling at a temperature of 400 °C revealed the formation of an ultrafine precipitation in the melted zone (Figure 17). The diffusion zone in hot-rolled Ti/Al joint has a significantly different character than in Mg/Al, consisting of a mixture of both materials, together with the ultrafine dispersion of TiAl_3_ intermetallic precipitates. This specific microstructure suggests that intermetallic phase forms a Ti/Al solid solution during hot rolling.

**Figure 18:**
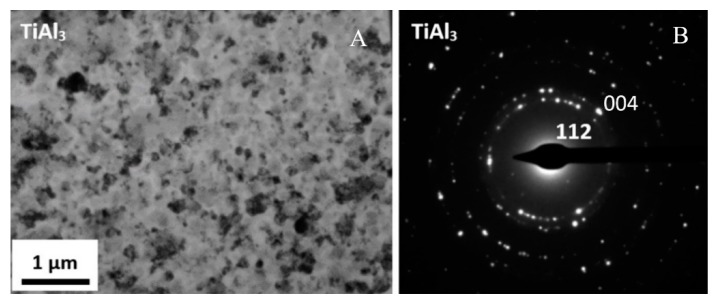
TEM image of AA1050/Ti6Al4V joint microstructure in hot-rolled state (**A**), with selected area diffraction patterns (SAED) (**B**). TEM lamella position in Figure 17.

The TiAl_3_ intermetallic phase is most likely from a thermodynamic point of view. In the case of Al/Ti, intermetallic growth is in the nature of precipitation rather than layered growth, like in the case of the Mg/Al interface. The presence of TiAl_3_ was confirmed by TEM observation and the results of the SAED electron diffraction technique (Figure 18). Investigations revealed ultrafine precipitates of the TiAl_3_. The size of the precipitates was approximately 100 nm.

## 4. Conclusions

The analysis of the results shows that explosive welding allows to obtain Mg/Al/Ti multilayer composite, characterized by lack of any imperfections in joints, such as delamination, voids or cracks. The application of the post-weld hot-rolling process results in the diffusion of elements under the influence of temperature and plastic deformation. Hot-rolling at 300 °C results in the highest reported level of strain hardening of AZ31 and AA2519 components. The curves obtained in the tensile test of mini-specimens allow one to draw a conclusion that post-weld hot rolling affects both joints in a different way. The Mg/Al interface shows an accordance of tensile strength and rolling temperature, with the highest value of 125 MPa reported for rolling at 400 °C. At the same time, the Al/Ti interface is characterized by significant differences in tensile strength, with 93 MPa value reported for sample in the as-welded state and 135 MPa for the sample after hot-rolling at 400 °C. The microstructure analysis revealed that hot-rolling affects the microstructure of both joints. In the case of the Mg/Al interface, the growth of continuous intermetallic layers and, for the Al/Ti interface, the precipitation of TiAl_3_ intermetallic from solid solution (melted zone), have been reported.

## Figures and Tables

**Table 1:** Chemical composition of the laminate components.

**AZ31**	**Al**	**Zn**	**Mn**	**Si**	**Cu**	**Ca**	**Fe**	**Mg**
	2.50<	0.60<	0.20	0.10	0.050	0.040	0.0050	Rest
**AA1050**	**Fe**	**Si**	**Cu**	**Mg**	**Mn**	**Zn**	**Ti**	**Al**
	0.4	0.25<	0.05<	0.18	0.05<	0.07<	0.05<	Rest
**AA2519**	**Fe**	**Si**	**Cu**	**Mg**	**Zr**	**Sc**	**Ti**	**Al**
	0.08	0.06	5.77	0.18	0.2	0.36	0.04	Rest
**Ti6Al4V**	**O**	**V**	**Al**	**Fe**	**H**	**C**	**N**	**Ti**
	<0.20	3.5	5.5	<0.30	<0.0015	<0.08	0.05<	Rest

**Table 2:** The designation of the samples.

Designation	State of the Sample
EXW	Mg/Al/Ti composite in the as-welded state
300C	Mg/Al/Ti composite subjected to the post-weld hot-rolling at 300 °C
350C	Mg/Al/Ti composite subjected to the post-weld hot-rolling at 350 °C
400C	Mg/Al/Ti composite subjected to the post-weld hot-rolling at 400 °C

**Table 3:** Tensile tests results with standard deviation.

	Ti6Al4V/AA1050/AA2519		AZ31/AA1050/AA2519	
	Rm (MPa)	Standard Deviation	Rm (MPa)	Standard Deviation
As-welded	101	7.15	114	6.22
Hot-rolled 300 °C	154	2.87	109	3.12
Hot-rolled 350 °C	132	3.50	115	3.30
Hot-rolled 400 °C	135	4.25	125	5.02
